# A peek behind the curtain: An integrative review of sexual harassment of nursing students on clinical placement

**DOI:** 10.1111/jocn.16600

**Published:** 2022-12-13

**Authors:** Ella Smith, Janice Gullick, Dawn Perez, Rochelle Einboden

**Affiliations:** ^1^ Susan Wakil School of Nursing and Midwifery The University of Sydney Camperdown New South Wales Australia; ^2^ Cardiac Device Notification Platform, eHealth NSW Chatswood New South Wales Australia; ^3^ School of Nursing and Midwifery Western Sydney University Penrith New South Wales Australia; ^4^ Endowed Research Chair in Nursing Care of Children, Youth, and their Families University of Ottawa, Faculty of Health Sciences, School of Nursing Children's Hospital of Eastern Ontario (CHEO) & CHEO Research Institute Ottawa Ontario Canada

**Keywords:** clinical placements, nursing students, sexual harassment, workplace violence

## Abstract

**Aims and Objectives:**

This integrative review aimed at systematically searching, analysing and synthesising multiple sources of evidence, to build a temporal understanding of nursing students' experiences of sexual harassment whilst on clinical placement, and to discuss the social context which enables this.

**Background:**

Sexual harassment is highly prevalent in workplaces globally. Contemporary social understandings contextualise sexual harassment as a significant form of interpersonal violence. This is the first literature review to go beyond prevalence to synthesise the experience, implications and responses to sexual harassment encountered by student nurses on clinical placement.

**Design:**

Whittemore and Knafl's (2005) integrative review methodology is used to structure a rigorous analysis and synthesis of evidence.

**Methods:**

The PRISMA checklist supported sound reporting of the search strategy. Three databases (CINAHL, Scopus and Medline) were searched using a Boolean strategy. Papers with a significant focus on sexual harassment of nursing students in the clinical setting were included with no limitation on publication date. Papers were excluded if they were not published in English or were only published as abstracts.

**Results:**

A synthesis of 26 papers demonstrated that sexual harassment has significant impacts on student nurses and the nursing profession. The intimacy of close body care, dominant social perceptions of nursing as women's work and the sexualisation of nurses increase student vulnerability to sexual harassment. Workplace power imbalances further exacerbate sexual harassment and shape responses to incidents.

**Conclusions:**

Sexual harassment of nursing students is widespread and impacts student well‐being and learning.

**Relevance to Clinical Practice:**

Education is a strong protective factor and should target students, clinicians, clinical facilitators and academics. Attention to workplace culture, and processes that support disclosure and reporting, is also required to meaningfully address the sexual harassment of nursing students.

**What does this paper contribute to the wider global clinical community?**

- A consideration of current evidence that describes sexual harassment of nursing students within contemporary social understandings of workplace violence.
- A conceptual discussion of the social contexts that allow and increase the likelihood of nursing students and nurses experiencing sexual harassment in the workplace.
- Synthesis of available evidence to offer practical recommendations for both practice and educational policy that may assist in decreasing the likelihood of the sexual harassment of nursing students.

## 1 INTRODUCTION

The Universal Declaration of Human Rights proposes that every person has the right to a safe workplace (United Nations, 1948). Although sexual harassment has existed throughout patriarchal societies, the term was not coined until feminist groups in the United States conceptualised it as an issue in the 1970s (McLaughin et al., 2012). As a result, sexual harassment became a prominent topic of legal discourse and has been considered an unlawful form of discrimination since the 1980s (Charlesworth et al., 2011; NOLO, 2022).

To guide this literature review, a synthesised, operational definition of sexual harassment was built through a combination of international sources as follows:

“Unwelcome, offensive, and undesirable sexual conduct connected to and impacting upon work, which may be verbal, visual, non‐verbal, or physical. Behaviour may be sexual in nature, or based on someone's sex, and causes the recipient to feel intimidated, uncomfortable, embarrassed, or humiliated” (Bronner et al., 2003; Cogin & Fish, 2009; Kim et al., 2018).

The impetus for this literature review was an emerging realisation that in our own academic institution (an Australian University), nursing students were raising concerns about sexual harassment on clinical placement including unclear reporting pathways, and perceived limitations in both the preparation of nursing students, and the preparation of clinical facilitators and nurse academics who teach and support them. This is against a background of very high lifetime prevalence of sexual harassment in the Australian community (85% of women and 57% of men) (AHRC, 2018) and nearly all (91%) of recent sexual harassment complaints in an Australian state being work‐related (Victorian Equal Opportunity & Human Rights Commission, 2019). This is echoed in a large UK survey of the general population where 29% of employed people experienced workplace sexual harassment in the past three months (Adams et al., 2020).

Internationally, women make up the majority of the nursing workforce comprising 88% in Australia and the United States (AACN, 2017; NMBA, 2021), 90% in the United Kingdom (Royal College of Nursing, 2020) and around 98% in China (Prosen, 2022). As sexual harassment is often directed towards females (Ganapathy, 2018; Lee et al., 2011), female‐dominated professions such as nursing may carry a higher burden of sexual harassment (Durana et al., 2018). An estimated 25% of nurses worldwide experience sexual harassment in the workplace, and this increases to 38.7% in English‐speaking countries including Australasia, Canada, the UK and the USA (Spector et al., 2014). Given that nursing students (also predominantly female) must undertake their clinical practicum in health workplace settings, it is logical that they may also be exposed to sexual harassment, and at a stage of their professional development where they may be ill‐equipped to manage such incidents or may not be familiar with reporting structures or sources of support. The justification of this literature review lies not only in the social context in which it is completed but also in the prevalence of sexual violence within the profession.

The contemporary social context also provides the impetus for this review. While sexual harassment might have been tolerated, overlooked or dismissed by nursing students, and professionals who facilitate their clinical learning in the past, it is increasingly understood as unacceptable. There are numerous reasons for people's new recognition and outrage towards the various forms of violence against women, particularly younger women who make up the majority of student nurses. The “#MeToo” movement, which gained international traction in 2017, combined with reports of sexual harassment/assault in parliaments internationally, and the indictment of multiple powerful men due to sexual misconduct, all contribute to a newfound global understanding that such behaviour is impermissible (Corey, 2017; Julios, 2022; Rossi, 2021). However, the nursing work undertaken by students occurs in a complex social milieu where close body work, and care of people in vulnerable situations, creates a certain intimacy not found in most professions. This literature review is timely because compared with a decade ago, recent social understandings of sexual harassment are unrecognisable, with a growing awareness of harassment as a significant form of interpersonal violence.

Throughout this review, nursing is discussed as a *practice* and the term *nursing work* is equally applicable to student nurses. Nursing students' work is performed under the supervision of nurses, but also mimics the role of a registered nurse; therefore, factors that influence the sexual harassment of nurses may well impact nursing students; perhaps more so. Although this review focuses on nursing students' experiences, the discussion of nursing more broadly is a vital backdrop to the social contexts in which nursing students experience sexual harassment in the clinical setting.

## 2 AIMS

This literature review aimed at systematically searching, analysing and synthesising relevant literature, to build a temporal understanding of nursing students' experiences of sexual harassment whilst on clinical placement, its prevalence, and to discuss the social context which allows for such interactions.

## 3 METHODS

An integrative review methodology (Whittemore & Knafl, 2005) has been chosen to structure a rigorous analysis and synthesis of data. This approach supported the inclusion of papers with a range of methodologies. The methods chapter of this review follows Whittemore and Knafl's five stages of review: *problem identification* (within the introduction), *literature search*, *data evaluation*, *data analysis* and *presentation*.

### 3.1 Literature search

#### 3.1.1 Search strategy

The PRISMA‐S checklist for reporting literature searches was used to promote transparency of the search process (Page et al., 2021). A search was conducted through the online databases Scopus, Cumulative Index to Nursing and Allied Health Literature (CINAHL) Complete and Medline. Both Scopus and CINAHL Complete were searched on the 1st of September 2021, and Medline was searched on September 13th, 2021, by one author (ES), and where inclusion eligibility was uncertain, articles were discussed and consensus was reached between all authors. Search terms were developed using keywords/synonyms from the aim. Using a Boolean strategy with variations based on database specifications the search terms “nursing students” AND “sexual harassment” OR “sexual violence” AND “clinical placement” were used (Table 1).

**TABLE 1:** Search terms

Concept	Synonyms	MeSH headings & keywords
nursing students	nursing student* student nurse* nursing pupil*	**MeSH headings** Students, Nursing Students, Nursing, Baccalaureate Students, Nursing, Practical
sexual harassment	sexual violence harassment, sexual	**MeSH heading** Sexual Harassment **Keyword** sexual violence
clinical placement	clinical practicum student placement	**MeSH term** Student Placement **Keyword** Clinical placement Clinical practicum

#### 3.1.2 Inclusion and exclusion criteria

Papers included were in the English language and referred to sexual harassment of nursing students in the title, aims or as part of their core findings to demonstrate a significant focus on sexual harassment of students. Literature reviews were included to better understand the scope of the issue and current knowledge on the topic. Grey literature such as case studies, discussion papers and opinion pieces was included to enable a thorough exploration of the social context, understanding and opinions towards sexual harassment. Limitations were not placed on the year of publication to capture a comprehensive understanding of the socio‐historical context of sexual harassment. This understanding of context is particularly relevant due to society's evolving views towards violence against women, and this decision allowed for analysis of change over time. Papers were excluded if they focussed on the sexual harassment of other healthcare students or professionals without the inclusion of nursing students. Published abstracts were excluded due to a lack of depth in content.

#### 3.1.3 Search outcomes

**FIGURE 1:**
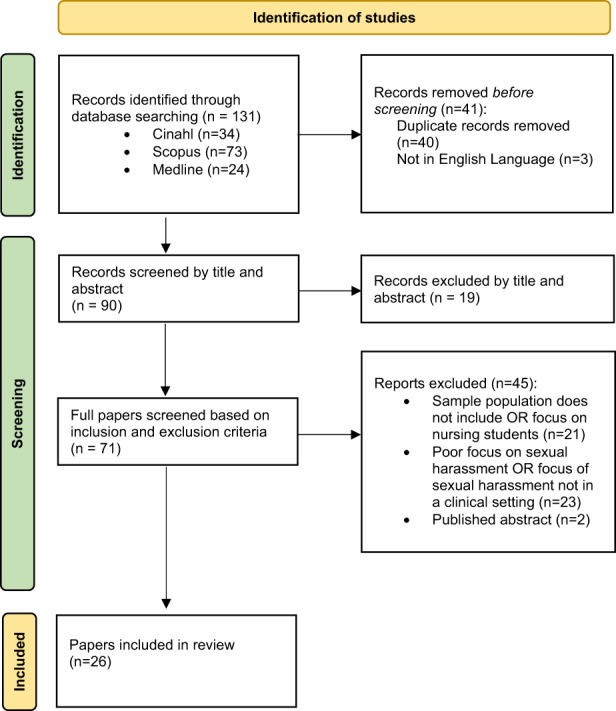
PRISMA diagram showing search process (Page et al., 2021)

Figure 1 represents the screening process using a PRISMA diagram (Page et al., 2021). Initially, there were 128 papers identified over the three databases. Duplicates (38) were manually identified and excluded. Three papers not in the English language were also excluded, leaving 87 papers for screening of titles and abstracts for relevance. A total of 70 papers identified to have the potential for inclusion were reviewed in full, and inclusion and exclusion criteria were applied. Following this process, 26 papers were included in the final review.

### 3.2 Data evaluation

Due to the diverse methods in the included publications, three quality appraisal tools were employed to assess rigour. The tool used for empirical studies was the mixed methods appraisal tool (MMAT; Hong et al., 2018). The tool used to assess the quality of grey literature was the Joanna Briggs Institute (JBI) checklist for text and opinion (McArthur et al., 2015). The tool used for literature reviews was the Critical Appraisal Skills Programme (CASP) Systematic Review Checklist (CASP, 2022).

Given the differing scoring systems between these tools, all quality appraisal scores were converted to a percentage score to manage comparison. The quality of papers did not affect their inclusion in this literature review, but evaluation of quality informs the reader of the strength of the evidence.

### 3.3 Data analysis

Whittemore and Knafl's (2005) 10 stages of data analysis guided the systematic and iterative interrogation of data. The stages of data analysis include the following:

1. *Noting patterns and themes*: papers were screened in full and common ideas in the data were coded and grouped.
2. *Seeing plausibility*: quality appraisal tools were employed to assess the quality of articles. Quality scores were converted to percentiles to allow quantification and comparison between papers of different study designs. Papers of lower quality were given less weight during analysis. Literature external to the review helped further situate findings and support reliability.
3. *Clustering*: literature was separated into empirical studies and grey literature and characteristics documented in two data extraction tables. Empirical research assisted in reporting of prevalence and overall sexual harassment experiences of both nurses and nursing students. Grey literature, including commentary, editorials and discussion papers, contributed to the understanding of opinion and social context.
4. *Counting*: main themes were tabulated to assist in counting the frequency of events/themes. The themes that were identified in many papers were given more consideration during analysis than minor themes.
5. *Making contrasts and comparisons*: the table built to assist in the “counting” stage of data analysis was analysed, and differences in data/opinions were noted. Publication dates were considered in relation to social context and opinions.
6. *Discerning common and unusual patterns*: minor themes were analysed with consideration of the overarching influential factors. Literature external from this review is cited to support minor themes and their contribution to understanding
7. *Subsuming particulars into the general*: several subthemes were conceptualised and collapsed into larger themes. For example, the theme of gender was collapsed into the larger theme of power imbalances. This provided structure to the analysis and central argument.
8. *Noting relations between variability*: where the variance between studies was identified (e.g. in date or country of publication), constructs that maintained their influence across these variables were considered (e.g. hierarchical position, gender, and role).
9. *Finding intervening factors*: during analysis, mechanisms that influenced experience such as environment, health systems and culture were considered.
10. *Building a logical chain of evidence*: a clear description of inclusion/exclusion criteria, study characteristics, study quality and transparency of methods is followed by a considered, referenced synthesis. Tables and figures support a sound presentation of the processes of analysis.

## 4 RESULTS

### 4.1 Study characteristics

**FIGURE 2:**
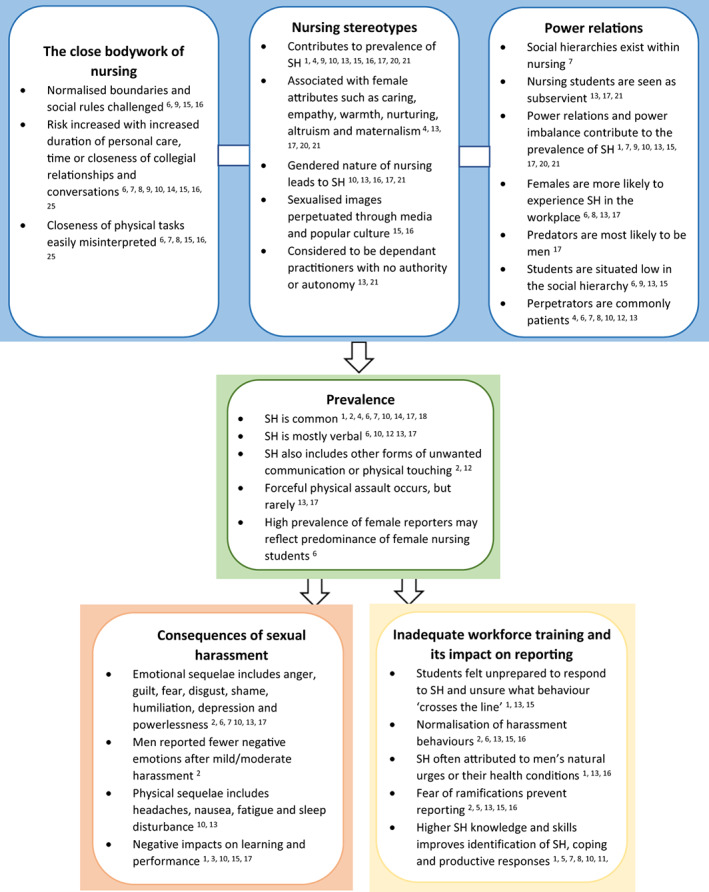
Superscript numbers denote article reference numbers in Tables 2 & 3

Two data extraction tables were developed. The first (Table 2) presents empirical studies and identifies the author, year, study design, aim, setting, sample, methods, quality score and findings of each included paper to assist in the reduction of data (Whittemore & Knafl, 2005). Where samples also included graduate nurses, or students of other healthcare specialties, only data that applied specifically to nursing students were presented. The second table (Table 3) presents grey literature and identifies the author, year, document type, purpose, jurisdiction, key content, quality appraisal score and main findings and recommendations for each paper. The use of these tables supported a comprehensive, systematic and transparent approach to the classification of data that supported analysis (Whittemore & Knafl, 2005). The final presentation of the findings of this review is an analytic discussion of themes and a summative figure mapping key findings to sources (Figure 2).

**TABLE 2:** Data extraction table for empirical studies

	Author	Study design	Aim	Setting	Sample	Methods	Quality score	Findings
1	Birks et al. (2018)	Descriptive, cross‐sectional survey study	To explore Australian nursing students' experiences of bullying and harassment whilst on clinical placements.	Online survey available to all Australian nursing students enrolled in baccalaureate nursing degrees.	Convenience sample of 884 Australian baccalaureate nursing students' surveys, 398 returned qualitative comments 88% female, mean age 26 yrs	Cross‐sectional online survey ‐ content analysis of open‐ended comments	MMAT: 100%	Examples of student sexual harassment included lewd conversations and sexual suggestions towards them or around them, direct homophobia and transphobia (including a gay student falsely accused of sexual harassment), stalking, being “hit on”, inappropriate touching by a preceptor, an educator rubbing himself in an aroused state against a student.
2	Bronner et al. (2003)	Descriptive, cross‐sectional survey study	To determine the prevalence of different types of sexual harassment, identify common perpetrators, explore the feelings evoked in nurses and nursing students and identify individual responses to such incidents.	Five medical centres in Israel	Convenience sample (487) 281 nurses, 206 nursing students 390 female, 97 male	Self‐report questionnaire ‐ Validated	MMAT: 100%	Workplace sexual harassment is common for nurses and nursing students. Compared to females, males showed significantly fewer negative emotions following mild or moderate sexual harassment (65% and 33% respectively). For 23% of nursing students who were harassed, the level was severe compared to 33% of nurses (*p* = .032) and students reported a median of two versus 3 types of sexual harassment (*p* = .006). Nurses responded assertively more often than students (35.8% vs. 25.6%) and females more than males. In response to mild or moderate harassment, the most common perpetrators were male patients (18–38%), followed by male physicians (10–30%) and male nurses (15–22%). Female perpetrators were uncommon, but female patients performed more severe types of harassment (10–15%) while female nurses performed more mild types of harassment (12–14%). Training in assertiveness, and education on appropriate sexual conduct is required for nurses and nursing students. Male students and nurses may be subjected more commonly to severe forms of sexual harassment, and often respond less assertively than their female counterparts.
3	Budden et al. (2017)	Descriptive, cross‐sectional survey study	To determine the incidence and type of bullying and harassment experienced by student nurses on clinical placement, the type of perpetrators, the impact on students and the management of reporting	Online survey available to all Australian nursing students enrolled in baccalaureate nursing degrees	888 Australian nursing students. 89% were female and median age 26 years.	Self‐report questionnaire ‐ Validated	MMAT: 80% Unable to assess risk of non‐response bias	Of 888 participants, 11.6% reported sexual harassment occurring often or sometimes, and 35% reported occasional sexual harassment. Types of harassment included sexist remarks (15%), suggestive sexual gestures (13%), inappropriate touching (11%), unwanted request for intimate physical contact (6.6%), and threat of sexual assault (1%). Reporting of other measured elements not separated from other types of harassment.
4	Çelebioĝlu et al. (2010)	Descriptive, cross‐sectional survey study	To determine nursing students' experiences of violence in clinical settings. Also aimed to explore the type of violence, effects of such interactions, and whether students often confronted this behaviour.	School of Nursing and School of Health, Ataurk University.	Convenience sample 380 nursing students gender not specified	Questionnaire	MMAT: 100%	Of 191 reported cases of violence 4.2% was sexual violence. In the 8 cases of sexual violence 3 perpetrators were patient/family, 3 were doctors and 2 were other staff. Education around communication, and responses to violence in the workplace should be implemented.
5	Celik and Bayraktar (2004)	Descriptive cross‐sectional survey study	To identify the frequency, type, source, and effects of abuse on nursing students. Also aimed to explore students coping strategies following abuse.	Nursing school in Ankara, Turkey	Convenience sample 225 nursing students Gender not specified	Questionnaire ‐ validated	MMAT: 100%	Abuse of nursing students was identified as a major issue with 53.3% of students having experienced sexual abuse. Sexual abuse is most often perpetrated by patients (32.5%) followed by patient relatives (30%) and faculty staff (9.2%). 22.7% of nursing students reported experiences of sexual harassment on placement. Examples were unwanted sexual jokes/conversation, being asked out, unwanted mail or phone contact, suggestive behaviours and touching or being shown someone's body in a sexual way. Sources of abuse were patients (32.5% < family (30% < physicians (28%) and faculty (9.2%). Training and education surrounding violence is suggested.
6	Chang et al. (2021)	Randomised controlled trial	To evaluate the effects of a clinical‐based sexual harassment prevention e‐book on nursing students' knowledge, prevention strategies, coping behaviours, and learning motivation	A university in central Taiwan	Stratified block randomisation 66 fourth‐year nursing students	Randomised controlled trial	MMAT: 80% (unable to assess whether outcome assessors are blinded to intervention)	A clinical‐based sexual harassment prevention e‐book was effective in significantly improving nursing students' prevention knowledge and competence in responding to sexual harassment. Coping behaviours were significantly higher in both groups after the intervention (but no difference between groups)
7	Chang et al. (2020)	Descriptive, cross‐sectional survey study	To report nursing students' experiences, knowledge, responses, and determinants of sexual harassment during clinical placement.	four universities in Central Taiwan	Convenience sample 291 senior nursing students 253 female, 38 male	Cross‐sectional survey using a self‐report questionnaire	MMAT: 100%	22.7% of participants had experienced sexual harassment during placement. Improved educational programmes are required to prevent incidents of sexual harassment towards nursing students and improve gender sensitivity among the population.
8	Cogin and Fish (2009)	Mixed method (quantitative survey & semi‐structured interviews)	To explore the prevalence of sexual harassment in nursing. Also aims to identify environmental factors that contribute to such incidents.	Eight Australian public hospitals in NSW and Victoria	Representative convenience sample (538 participants) 287 nurses, 251 nursing studentsl442 female, 96 male	mixed methods: questionnaire & semi‐structured interviews	MMAT: 70% (poor integration of qualitative and quantitative components; Response rate of 21.6%)	60% of female participants and 30% of male participants reported experiencing an incident of sexual harassment in the 2 years prior to the interview/survey with a higher prevalence for students (68%) versus graduate nurses (45%). Nurses' perceptions surrounding leadership styles of their managers are an important variable in sexual harassment. An unbalanced gender ratio in the workplace contributes to the high incidence of sexual harassment in nursing. Sexual harassment directed to nurses from patients are less likely to instil feelings of intimidation, humiliation or embarrassment compared to the same behaviour from physicians or colleagues.
9	El‐Ganzory et al. (2014)	Pre‐post intervention survey study	to investigate the effect of an educational guideline programme on internship nursing students facing sexual harassment behaviours.	Ain Shams University Hospital (Egypt)	Purposive sample 60 nursing students 52 female, 8 male	Structured questionnaire	MMAT: 60% (unable to assess risk of nonresponse bias) Conflicting data on age reported between table and text	Patients were the perpetrator in 71% of cases followed by relatives and physicians. Only 28.3% reported the harassment. None had received education on sexual harassment prior to the intervention. This educational intervention significantly improved student nurses' knowledge of, emotional reactions to, and coping mechanisms, related to incidents of sexual harassment.
10	Ferns and Meerabeau (2008)	Descriptive, cross‐sectional survey study	To explore the nature, degree, prevalence and perpetrators of verbal abuse towards nursing students during clinical placements.	One pre‐registration nursing programme in England.	Convenience sample 114 nursing students 91 female, 14 male	Questionnaires ‐ Validated	MMAT: 100%	64.7% of general verbal abuse (inclusive of sexual verbal abuse) was perpetrated by patients, followed by visitors/relatives (15.7%), & colleagues (19.6%). Quantitative results did not differentiate sexual from other abuse, however, qualitative data exemplified distressing sexual harassment of students. Education, support, and current policies surrounding verbal abuse are not efficient in preparing nursing students, and should be better implemented.
11	Finnis and Robbins (1994).	Quantitative & qualitative survey	Outlines the extent, nature, common perpetrators and circumstances of sexual harassment of nursing students and nurses. Also aims to explore the relationship between sex role identity and assertiveness to sexual harassment.	One university (location not specified) Published in the United Kingdom	Convenience (91) 65 nurses, 26 students 87 female, 5 male (Nurses asked to reflect on harassment experiences as students)	Piloted questionnaire Theoretical lens	MMAT: 100%	77% reported experiencing sexual harassment as students during clinical placement. Both physical and verbal sexual harassment was highly prevalent for nurses and nursing students, especially females with the perpetrator being male in 92% of cases. Most common perpetrators for students were patients and doctors. Sexual harassment caused physical responses such as sleep disturbance, and headaches, anger, disgust, annoyance, resentment, and embarrassment.
12	Hallett et al. (2021)	Convergent mixed methods	To explore the prevalence of aggression towards nursing students during placement and report the rates and experiences of reporting the same. Aims to provide a thorough understanding of students' experiences of workplace violence.	Two universities in the United Kingdom	Convenience sample 129 Pre‐registration nursing students 120 female, 7 male	Mixed methods: cross‐sectional survey and qualitative focus group	MMAT: 100%	Nursing students commonly experience many types of aggression whilst on placement. One in three (39.5%) of students had been harassed sexually in the previous year, with nearly one‐third experiencing four or more incidents. Around half of sexual harassment cases were reported. Extended research into long‐term outcomes and effects of aggression as well as targeted educational programmes for nursing students would improve preparation for these experiences.
13	Kettl et al. (1993)	Pre‐post intervention survey study	To determine the prevalence of sexual harassment towards health care students whilst on clinical placement at a psychiatric hospital	3 Universities (locations not specified) Published in Pennsylvania	Convenience sample 158 nursing and occupational therapy students 151 female, 7 male	Quantitative questionnaire	MMAT: 80% unable to assess risk for nonresponse bias)	Nursing students were analysed separately from occupational therapy students with no significant differences in results. Harassment took the form of verbal innuendo (40%), verbal request for sex (22%) minor physical sexual assault 18%) and forceful sexual assault (16%). Common responses to sexual harassment identified include ignoring the behaviour (39%), verbal redirection, limit setting, education about the relationship, and physical intervention. 49% had received education about handling sexual harassment, yet 86% felt such training was necessary.
14	Lee et al. (2011)	Qualitative descriptive study	Identify Korean nursing students' experiences and perceptions of sexual harassment during clinical placement	12 Nursing colleges in Korea	Convenience sample 542 nursing students only single, female students sought for inclusion)	self‐report questionnaire	MMAT: 100%	Nursing students are a risk group for sexual harassment, with 52% of students indicating they had experienced at least one incident. The perpetrator was found to be male in 97.9% of cases (NB: there were no male students recruited). Psychiatric wards were the highest venue of prevalence (67%). 60% had received training in sexual harassment in the past and 88% believed training was necessary to create a safe educational space for clinical placement.
15	Magnavita and Heponiemi (2011)	Descriptive, cross‐sectional survey study	To better understand the characteristics and effects of violence towards nurses and nursing students, compare the differences between the two groups, and build preventative measures.	Three Italian universities, and one general Italian hospital	Convenience 275 nurses, 346 students 461 female, 155 male	Retrospective survey	MMAT: 100%	20% of students reported experiencing sexual harassment in the 12 months prior to the study compared to 37% of nurses. Majority of nurse and student victims of sexual harassment and stalking were female (82–88%) Protective measures are required to manage violence in clinical practice towards nurses and nursing students from both patients and colleagues.
16	Kim et al. (2018)	Interpretive phenomenological study	To examine nursing students experience of sexual harassment during clinical placements.	Two universities in South Korea	Purposive snowball sampling 13 nursing students 11 female, 2 male	Individual in‐depth interviews	MMAT: 100%	Twelve themes included: “unprepared to respond”, “lack of education”, “unsure about when behaviour crosses the line”, “power differential for nursing students”, “balancing self‐preservation with obligations to patients”, “shame”, “feeling responsible for not being able to prevent the harassment”, “impact on patient care”, “fear of what might have happened”, “fear of repercussions”, “long term impact”, and “peer support”. Nursing students struggled to balance the obligation to care with feelings of vulnerability due to sexual harassment and negative emotions arising from sexual harassment Support in recognition and response to sexual harassment is vital to ensure quality learning and quality care.
17	Seed (1995)	Longitudinal grounded theory study	To narrate the experiences of student nurses whilst providing intimate care in the clinical setting over the course of their studies.	Multiple clinical placement settings of students over a 3‐year period throughout the United Kingdom	Not stated, query theoretical sampling of 23 student nurses 20 female, 3 male	Participant observation & interviews Grounded theory	MMAT: 80% (does not explicitly state aim of paper)	Students found giving intimate care to “different” (gendered) bodies stressful. They described having to consciously view them as “patients” rather than “men” (or for male students, women). Perpetrators were difficult to avoid at work. Female students were cast in the role of “available female”. Female students were criticised for talking to younger male patients and were redirected to tasks such as cleaning so that “establishment of nurse patient relationships was discouraged by systems of work”. Sexual harassment was normalised socially as “just something men do”. Some students hid their nursing identity outside of work because of a “sexy nurse” stereotype promoted in popular culture and tabloid media. The caring of male students was seen as atypical in that “caring is ‘given to’ women and that ‘not caring’ becomes the defining characteristic of manhood. Humour and ignoring the incident was used as a coping mechanism.
18	Tollstern et al. (2020)	Descriptive, cross‐sectional survey study	To identify the prevalence, subtypes, and consequences of sexual harassment towards nurses and nursing students at a hospital in Tanzania.	A regional university hospital in Tanzania	Convenience sample 97 nurses and 100 nursing students 133 female, 64 male	Cross‐sectional questionnaire ‐ Validated	MMAT: 100%	10% of participants had been subjected to sexual harassment in the workplace. No significant difference between nurses and students in frequency of different types of sexual harassment. 1.4% of students had experienced severe sexual assault (trying to have sex with them). When including by proxy victims, the prevalence of sexual harassment rose to 36.5%. The most frequent forms were ‘sexual jokes and comments’, and ‘unwelcome hugging or kissing’. Females (73.7%) and students (11% versus 8%) experienced higher rates of sexual harassment. Perpetrators were mostly male physicians. Over 30% of participants reported feeling depressed and uncomfortable returning to work, and 40% stated they felt angry and afraid.
19	Zeng et al. (2019)	Systematic review and meta‐analysis of observational studies	To analyse the prevalence of sexual harassment of nurses and nursing students in China	China	Reviewed literature from English & Chinese databases: **English**: Pubmed, EMBASE, PsychINFO, Web of Science, & OVID **Chinese**: China National Knowledge Internet, WanFang, SinoMed, & Chinese VIP information	Meta‐analysis	CASP systematic review checklist 77.7% (clearly focussed issue but no research question. Not all outcomes considered. Question 10 re benefits excluded as no intervention)	Sexual harassment is highly prevalent for nurses (7.5%) and nursing students (7.2%) in China, with an estimated 129,600 students being possibly subjected. Preventative workplace methods should be developed and implemented.

**TABLE 3:** Data extraction table for grey literature

	Author	Document type	Purpose	Jurisdiction	Key content	Quality measure & score: JBI checklist for text & opinion (McArthur et al., 2015)	Findings/recommendations
20	Castledine (2002)	Case study	Educate nurses about how sexual misconduct and harassment breaches the nursing code of conduct, and highlight the process of reprimanding and investigating staff reported for the same.	United Kingdom	Breaches in code of conduct Sexual harassment of nursing students Investigating staff Consequences to health professional perpetrators of sexual harassment	83.33% (incongruence with literature/sources not logically defended)	Sexual harassment of nursing students from other healthcare professionals breaches the code of professional conduct. Perpetrators reported for sexual misconduct will be judged based on the whole of their practice, not just the incident reported.
21	Chapman (1993)	Commentary	To educate nurses and students in New Zealand of legal avenues for reporting incidents of workplace sexual harassment.	New Zealand	Defines sexual harassment. Highlights legal avenues for reporting in sexual harassment in the clinical setting throughout New Zealand.	83.33% (incongruence with literature/sources not logically defended)	Health settings hold a responsibility to ensure a safe work environment with appropriate avenues for reporting incidents of sexual harassment for employees and students. Nursing education providers should include education about sexual harassment in the nursing curriculum.
22	Ganapathy (2018)	Discussion paper	To build an understanding of the nature, types, motivations, and methods of sexual harassment. Also, to better understand common responses, and current perpetuated myths about nurses that perpetuate the behaviours.	Not reported Published in India	Draws on sources such as media, and current legislation to discuss impact of sexual harassment.	73.33% (unable to ascertain analytical process of opinions; quotes witnesses without describing source)	The media's portrayal of nurses as well as poor policy, legal support, and negative stereotypes contribute to the high prevalence of sexual harassment for nurses and nursing students. In response to sexual harassment, many nurses and students either do nothing or pretend they have not experienced or witnessed sexual harassment. Unequal power relationships within hospitals and academia increase nursing students' vulnerability to harassment. Myths and stereotypes about nurses as dependent, sexy, nurturing, intimate, and available are tools of oppression. Open discussion, and education is needed to address the social, cultural, and environmental issues identified with sexual harassment.
23	Patrick (2020)	Opinion piece	To debate the normalisation of sexual harassment in nursing	United Kingdom	Uses media sources to build an argument against the sexual harassment of nurses. Poor culture and support in the profession	66.66% (no analytical process, no reference to extant literature)	In order to denounce sexual harassment in the workplace, the healthcare workforce should better unite and support one another.
24	Perry (1990)	Editorial	To encourage nursing students to “speak out” against sexual harassment, and not tolerate the behaviour.	United Kingdom	Sexual harassment contributes to a hostile work environment and is not appropriate. Common responses and consolations to incidents of sexual harassment. Fighting sexual harassment is everyone's responsibility.	50% (logic of opinion unclear; no reference to extant literature; incongruence with literature/sources not logically defended).	Sexual harassment contributes to a poor work environment and culture. It is a person's right to a safe work environment, and to decrease sexual harassment, student nurses should assert themselves and report incidents.
25	Trueland (2013)	Case study/discussion paper	Improve awareness of the types of harassment nurses and nursing students face in the workplace. Considers whether the response from institutions has changed.	United Kingdom	Examples of sexual harassment Hierarchy within hospitals, Nursing students' experiences of placement, Education & importance of reporting.	50% (author's expertise not clear; no reference to extant literature; incongruence with literature/sources not logically defended)	The hierarchy within healthcare protects some and leaves others vulnerable. Students who feel powerless are less likely to assertively respond to inappropriate behaviour. States that reports/allegations of sexual harassment would be taken seriously.
26	Wolfe (1996)	Case study/Opinion piece	Improve audiences' understanding of the context in which sexual harassment occurs towards nurses in the workplace, reporting systems in place, and employers' responsibilities.	United States	Power influences on sexual harassment, Nature of nursing profession increasing risk of sexual harassment, Legal avenues of reporting	83.33% (incongruence with literature/sources not logically defended)	The best response to sexual harassment is to assert boundaries and gain support from your union/managers. Documentation of sexual harassment incidents is important for later reporting. Employers should have written policies and formal reporting procedures in place, and promptly investigate reported incidents.

The papers included had a publication range from 1990 to 2021 (see Tables 2 and 3). Of those papers that recruited participants, the sample size ranged from *n* = 13 to *n* = 888 with variability explained by research design (e.g. qualitative versus quantitative research). Of the 26 papers identified for inclusion, the majority (eight) were from the United Kingdom. There were five papers from East Asia, four from the United States, three from the Middle East and three from Australia. There was one paper each from New Zealand, South Asia, Europe, Egypt and East Africa (see Tables 2 and 3).

### 4.2 Quality appraisal

While the decision was made not to exclude papers based on quality, there were no papers that scored less than a 50% quality score using any tool. Among the 12 stand‐alone survey studies, nine were of high quality with a 100% MMAT score. The exceptions were three survey studies that received MMAT score reductions as it was not possible to assess the risk of nonresponse bias (80% for Kettl et al. (1993) and Budden et al. (2017); 60% for El‐Ganzory et al. (2014)). Additionally, the El‐Ganzory paper had conflicting data presented between table and text for age, and so this data were not included in the analysis. Of the two mixed methods studies, one received a reduction in MMAT score (70%) due to poor integration of qualitative and quantitative results and a low response rate of 21% (Cogin & Fish, 2009). The one randomised controlled trial (Chang et al., 2021) received a MMAT score of 80% as we were unable to assess whether outcome assessors were blinded to the intervention. Of the three qualitative studies, a grounded theory study (Seed, 1995) received a MMAT score of 80% because the aim of the study was not clearly stated. The one systematic review (Zeng et al., 2019) received a CASP score of 77.7% because, while there was a clearly focussed issue explained, there was no research question and not all outcomes were considered.

The grey literature was generally of lower quality with JBI scores between 50 and 83%. Common limitations included the following: incongruence with literature sources not logically defended (Castledine, 2002; Chapman, 1993; Perry, 1990; Trueland, 2013; Wolfe, 1996), lack of reference to extant literature (Patrick, 2020; Perry, 1990; Trueland, 2013), lack of a clear analytic process or clear logic in the opinion (Ganapathy, 2018; Patrick, 2020; Perry, 1990)—including quoting people without describing the source (Ganapathy, 2018) and the authors' expertise not made clear (Trueland, 2013). The individual quality scores and their rationale are tabled for each paper within a separate column (Tables 2 and 3).

### 4.3 Presentation

Nursing students' experiences of sexual harassment whilst on clinical placement were shaped by the underlying nature and social context within which nursing exists (*the close body work of nursing*, *nursing stereotypes* and *power relations*), the occurrence of sexual harassment (*prevalence*) and the outcomes following sexual harassment (*consequences of sexual harassment, inadequate workforce training, and their impact on reporting*; Figure 2).

### 4.4 Prevalence

Internationally, nursing students experience sexual harassment, with 7.2%–68% reporting an episode of sexual harassment during clinical placements at some point throughout their studies (Birks et al., 2018; Bronner et al., 2003; Budden et al., 2017; Celik & Bayraktar, 2004; Chang et al., 2020; Finnis & Robbins, 1994; Magnavita & Heponiemi, 2011; Zeng et al., 2019). There is variability in the proportion of student nurses versus graduate nurses experiencing harassment with some studies reporting a higher prevalence among students (Cogin & Fish, 2009; Tollstern et al., 2020), and others reporting a higher prevalence among graduate nurses (Bronner et al., 2003; Magnavita & Heponiemi, 2011), and one reporting no difference in prevalence (Zeng et al., 2019).

The nature of harassment was predominantly verbal (41%–62%) (e.g. lewd jokes or conversation, unwanted advances, sexualised remarks or insults about body or clothing) (Budden et al., 2017; Chang et al., 2020; Finnis & Robbins, 1994; Kettl et al., 1993; Lee et al., 2011; Tollstern et al., 2020), although gesturing, leering, touching, unwanted hugging or kissing, unwanted phone calls or mail, stalking, touching or exposing oneself sexually or attempting to have sex, were also widely reported. Forceful physical sexual assault was reported at between 0.9% and 2.6% of surveyed students (Lee et al., 2011; Tollstern et al., 2020), but reports were as high as 16–23% of students who were harassed (Bronner et al., 2003; Kettl et al., 1993). In one study, men were significantly more likely than women to receive severe forms of sexual harassment (either being forced to touch someone or attempts to have sex with them) (35% versus 26%) (Bronner et al., 2003).

### 4.5 Consequences of sexual harassment

Common emotional responses of nursing students to incidents of sexual harassment include anger, guilt, fear, disgust, embarrassment, humiliation, shame, depression and feelings of powerlessness (Bronner et al., 2003; Chang et al., 2020; Cogin & Fish, 2009; Finnis & Robbins, 1994; Lee et al., 2011; Tollstern et al., 2020). Compared with females, males showed significantly fewer negative emotions following mild or moderate sexual harassment (65% and 33%, respectively) (Bronner et al., 2003). Students who experienced sexual harassment not only experienced psychological impacts but also experienced physical effects such as headaches, nausea, general fatigue and sleep disturbances (Finnis & Robbins, 1994; Lee et al., 2011).

Sexual harassment has a substantial negative impact on nursing practice and work performance (Birks et al., 2018; Çelebioĝlu et al., 2010; Celik & Bayraktar, 2004; Chapman, 1993; Finnis & Robbins, 1994; Hallett et al., 2021; Kim et al., 2018; Perry, 1990; Tollstern et al., 2020). Important long‐term impacts on the care environment where students complete clinical placements were reported, with possible detriment to student learning and experience. These included high attrition and nursing staff turnover, increased job strain and decreased quality of care (Birks et al., 2018; Ferns & Meerabeau, 2008; Lee et al., 2011; Magnavita & Heponiemi, 2011). For students who are sexually harassed, substantial impacts include increased difficulty concentrating on practice or studies, absenteeism, reduction in confidence, loss of motivation, low job satisfaction, and low self‐esteem, and increased difficulty for students socialising within, and integrating into, the nursing profession (Finnis & Robbins, 1994; Kim et al., 2018; Tollstern et al., 2020). Such interactions lead to increased attrition from nursing programmes and increased levels of burnout (Birks et al., 2018; Çelebioĝlu et al., 2010).

### 4.6 Nursing stereotypes

The contribution of nursing myths/stereotypes to the high rates of sexual harassment of student nurses, and in nursing generally, was discussed in 11 of the 26 papers (Birks et al., 2018; Celik & Bayraktar, 2004; Chapman, 1993; Ferns & Meerabeau, 2008; Finnis & Robbins, 1994; Ganapathy, 2018; Kim et al., 2018; Lee et al., 2011; Seed, 1995; Tollstern et al., 2020). In explaining the sexual harassment of student nurses, the profession's long association with stereotypical feminine constructs of care was related to the expectation that nurses are caring, empathetic and warm (Celik & Bayraktar, 2004; Lee et al., 2011; Tollstern et al., 2020). This construction of nursing care as nurturing, altruistic and maternal may be related to the profession's religious roots. Registered nurses are still often referred to as “sister”, giving expectations of the trustworthy sibling or motherly role nurses are assumed to meet (Ganapathy, 2018; Lee et al., 2011). The negative consequences of these stereotypes for students relate to the expectations they create, including that they are readily available and willing to please at the expense of self, as a mother would be for their child (Celik & Bayraktar, 2004; Chapman, 1993; Ganapathy, 2018; Lee et al., 2011; Tollstern et al., 2020).

A second derogatory stereotype still strongly perpetuated is the sexual availability of nursing students (Ferns & Meerabeau, 2008; Finnis & Robbins, 1994; Ganapathy, 2018; Kim et al., 2018; Lee et al., 2011; Seed, 1995). The sexualisation of the nursing profession is a long‐standing problem for nursing students. For example, a student participant in a 1995 study described an image in a nursing magazine of students wearing “nothing but nurses' hats” (Seed, 1995, p. 1140). Seed's (1995) student participants referred to other examples of the sexualised imagery of nurses in low‐cut shirts and short dresses throughout newspapers and television programmes. More recently, Kim et al. (2018) describe how nurses are often stereotyped as “sex objects” (p. 379) and that this contributes to student nurse sexual harassment.

The conceptualisation of nursing as feminine is both generative of, and reproduced by, its workforce. The majority of nursing students identify as female, and sexual harassment is more prevalent for women (Ganapathy, 2018; Lee et al., 2011), increasing nursing students' risk of sexual harassment than professions with a higher proportion of men (Finnis & Robbins, 1994; Ganapathy, 2018; Lee et al., 2011; Seed, 1995). Although the sexualisation of nurses is gendered, male students also face damaging stereotypes that contribute to a high prevalence of sexual harassment. These include assumptions that males who wish to become nurses must be either gay or have joined the profession to meet women (Lee et al., 2011; Seed, 1995; Tollstern et al., 2020). These myths not only affect society's perception of nurses in general but alos effects responses from students towards sexual harassment.

The age of some studies, and the culture in which they were undertaken, may have had some influence on the stereotypes projected. An example of temporal influences on the student experience is the reference to a widely distributed politically incorrect comedy (the “Carry On” movies made between 1958 and 1992) (Seed, 1995) which would likely not be tolerated in contemporary media or society. An example of a possible cultural influence is found in a Korean study where student participants thought assertively responding to sexual harassment by a patient may impede patient care, and damage rapport and they considered it inappropriate for students to appear upset in the hospital setting (Kim et al., 2018). A second Korean study only sought to include unmarried females in their sample of 542 student nurses suggesting sexual harassment is seen as a single woman's problem (Lee et al., 2011).

Another stereotype of nursing is the long‐held assumption that nurses are dependent practitioners, working to please the doctor and without decision‐making authority and autonomy in their practice; a point that is intensified for student nurses (Ganapathy, 2018; Lee et al., 2011).

### 4.7 Power relations

The conceptualisation of nurses as subservient locates student nurse sexual harassment within hierarchical relations of power in the workplace (Ganapathy, 2018; Lee et al., 2011; Tollstern et al., 2020). The nursing profession is situated within social hierarchies, influenced by status and gender (Cogin & Fish, 2009). Ten of 26 papers referred to the relationship between sexual harassment and power imbalance (Birks et al., 2018; Chang et al., 2020; Chapman, 1993; Cogin & Fish, 2009; Ferns & Meerabeau, 2008; Finnis & Robbins, 1994; Ganapathy, 2018; Kim et al., 2018; Lee et al., 2011; Tollstern et al., 2020). Sexual harassment is framed as an expression of dominance, and a person's desire to gain or maintain power over another person (Finnis & Robbins, 1994; Ganapathy, 2018). The power imbalance is further attributed to both the perpetrator's privilege within the health workplace hierarchy and to “physical strength” (Chapman, 1993; Tollstern et al., 2020).

Gender was shown to further exacerbate the power inequities for student nurses: nursing students who experienced sexual harassment were overwhelmingly female (74–97%) and this reflected the higher number of female student participants. (Chang et al., 2020; El‐Ganzory et al., 2014; Tollstern et al., 2020). Only one study reported frequency within student gender groups with 23.3% of female students versus 18% of male students experiencing sexual harassment (El‐Ganzory et al., 2014).

Perpetrators were predominantly male (75–98%) (Chang et al., 2020; Finnis & Robbins, 1994; Lee et al., 2011; Tollstern et al., 2020); and predominantly patients, typically followed by family/friends and physicians (Bronner et al., 2003; Celik & Bayraktar, 2004; Chang et al., 2020; El‐Ganzory et al., 2014; Finnis & Robbins, 1994; Hallett et al., 2021; Lee et al., 2011). The exception was a study from sub‐Saharan Africa where the most common perpetrators were male physicians (43%), who harassed student nurses at a higher frequency than nurses (Tollstern et al., 2020). Culture and gender are related and important to power relations, with culture influencing the degree to which stereotypical expressions of masculinity and femininity are valued. For example, in cultures with a high masculinity index, women are more likely to tolerate sexually aggressive behaviour than in societies with a lower encumbrance of gender roles and values (Tollstern et al., 2020).

As patients are identified as the most common perpetrators of sexual harassment towards nursing students, it is, therefore, important to consider the relations of power between them (Celik & Bayraktar, 2004; Chang et al., 2020; Cogin & Fish, 2009; Finnis & Robbins, 1994). During clinical placements, nursing students are assessed on their ability to communicate and build rapport with patients (Lee et al., 2011). This creates significant pressure on students not to disrupt or damage therapeutic relationships. Although not direct evaluators of student performance, patients have potential power, with any complaints against students negatively impacting clinical practice assessment (Lee et al., 2011). This likely explains why the most common response of students to sexual harassment is to do nothing or pretend not to see or hear the abuse (Celik & Bayraktar, 2004; Ganapathy, 2018; Kettl et al., 1993), or to take sick leave (El‐Ganzory et al., 2014).

Other reasons locate nursing students on placement as low in the social hierarchy: they are not permanent employees, they require supervision and are inexperienced clinicians (Chang et al., 2020; Lee et al., 2011). The title of “student” itself announces the inexperience of nursing students on clinical placement (Ferns & Meerabeau, 2008). This low hierarchical positioning affects both the student's perceptions of themselves and the perceptions of others (Budden et al., 2017; Kim et al., 2018).

### 4.8 The close bodywork of nursing

Eight of 26 papers recognised that the close body work involved in nursing influences the risk and prevalence of sexual harassment (Chang et al., 2020; Cogin & Fish, 2009; El‐Ganzory et al., 2014; Ferns & Meerabeau, 2008; Kim et al., 2018; Magnavita & Heponiemi, 2011; Seed, 1995; Wolfe, 1996). The nursing student's close work with patients often involves breaking “social rules” and normalised boundaries as they assist with intimate tasks such as showering, toileting and tasks of daily living (Chang et al., 2020; Ferns & Meerabeau, 2008; Kim et al., 2018; Seed, 1995).

Sexual harassment has been found to occur most often during personal care (Finnis & Robbins, 1994) and the duration of time spent independently providing bedside care increases the risk of sexual harassment (El‐Ganzory et al., 2014; Magnavita & Heponiemi, 2011). Nursing students are encouraged to build a therapeutic relationship with patients, yet they are new to navigating the professional role where the boundaries are different, and the closeness of the relationship and the physical closeness of caring tasks are easily misinterpreted by some as sexual (Chang et al., 2020; Cogin & Fish, 2009; El‐Ganzory et al., 2014; Kim et al., 2018; Seed, 1995; Wolfe, 1996). Similarly, among staff, intimate conversations about sexuality and bodies are common, and often necessary for patient care (Wolfe, 1996). These common topics of conversation and close collegial relationships increase the risk of sexual harassment between staff (Wolfe, 1996). Because current nursing education places emphasis on nurses' caring role, students are hesitant to respond assertively to patients who exhibit sexually aggressive behaviour (Chang et al., 2020).

### 4.9 Inadequate workforce training and its impact on reporting

A need for increased education to reduce, and manage the prevalence and impact of sexual harassment for nursing students within the clinical setting was identified in 18 of the 26 papers (Birks et al., 2018; Bronner et al., 2003; Budden et al., 2017; Çelebioĝlu et al., 2010; Celik & Bayraktar, 2004; Chang et al., 2021; Chapman, 1993; Cogin & Fish, 2009; El‐Ganzory et al., 2014; Finnis & Robbins, 1994; Hallett et al., 2021; Kettl et al., 1993; Kim et al., 2018; Lee et al., 2011; Magnavita & Heponiemi, 2011; Seed, 1995; Tollstern et al., 2020; Zeng et al., 2019).

The evidence of nursing students' poor identification of their experiences as sexual harassment has been used by authors to argue for improved training. In a phenomenological study from Korea (Kim et al., 2018), qualitative themes of being “unprepared to respond”, having a “lack of education”, and being “unsure about when behaviour crosses the line” (Kim et al., 2018) are ideas that are echoed throughout the included literature. In another Korean study, before sexual harassment training, only 17.9% of nursing student participants were sure they had experienced sexual harassment. When the same students were offered a checklist of sexual harassment behaviours, those who claimed an experience of sexual harassment increased to 52% (Lee et al., 2011). These discrepancies support the findings of Kim et al. (2018) that nursing students have difficulty identifying sexual harassment.

Students commonly attribute causes of sexual harassment from patients to health conditions, gender differences or the sexual impulse and instincts of men (Lee et al., 2011; Seed, 1995). These common beliefs suggest that sexual harassment is due to male biological urges, further perpetuating the belief that such treatment is to be tolerated and to be expected (Birks et al., 2018; Lee et al., 2011). An older study noted that in the early stages of their studies, nursing students were less likely to confront and more likely to excuse patients who sexually harassed them, based on the patients' vulnerabilities due to illness (Seed, 1995).

The naturalisation of men's violence by way of sexual harassment translates to the low incidence of students reporting incidents in the workplace. The low incidence of reporting is also complicated by widespread ignorance and social acceptance of sexual harassment. Victims are required to make judgements about the validity of their experience of violation and are responsible to address the perpetrator's behaviour (Kim et al., 2018; Lee et al., 2011). Students' perception of the workplace hierarchy affects their willingness and desire to report incidents of sexual harassment, as they feel poorly positioned to respond assertively and feel an obligation to preserve the relationship between their places of study and hospitals (Kim et al., 2018). This lack of knowledge results in students having a poor understanding of how reporting will (or will not) impact their grades and relationships, thus further increasing the hesitancy to report (Kim et al., 2018; Lee et al., 2011). Further reasons identified for not reporting incidents of sexual harassment include fear of being disadvantaged during placement, being considered responsible for the interaction and the expectation that little will be done in response to the report (Kim et al., 2018; Lee et al., 2011; Seed, 1995). This may explain why students in one study were less likely than graduate nurses to respond assertively to sexual harassment (25.6% versus 35.8%) (Bronner et al., 2003). For example, in response to incidents where the perpetrator attempted to have sex, nurses responded assertively 55.6% of the time, compared with 25% of students (Bronner et al., 2003). However, female students were more likely than males to respond assertively when sexually harassed (Bronner et al., 2003; Chang et al., 2020).

While more passive responses to incidents of sexual harassment include ignoring the behaviour (or avoiding the patient), using dismissive humour, and verbal redirection, there were also more proactive responses by students such as education about professional boundaries, and physical intervention (Bronner et al., 2003; Çelebioĝlu et al., 2010; Kettl et al., 1993). Three of 26 papers across the historical spectrum reported that students who were further in their degree and had previous gender‐related education or specific education towards sexual harassment, were more likely to identify incidents and respond more assertively (Chang et al., 2021; El‐Ganzory et al., 2014; Seed, 1995). For example, a Taiwanese study found that nursing students who had completed a gender‐related course were 1.86 times more likely to have an awareness of the possibility of sexual harassment (Chang et al., 2020). Greater knowledge and skills related to sexual harassment, and the students' growing sense of professional identity through their training enabled them to develop improved coping mechanisms, identify risks, and respond to sexual harassment in more productive ways (Chang et al., 2021; El‐Ganzory et al., 2014; Seed, 1995).

Despite the call for education being the strongest finding across the literature, many universities do not have a formal curriculum on responding to, or coping with, sexual harassment before clinical placement. Of the 60 students involved in an Egyptian study, 100% stated they had not received any training on sexual harassment (El‐Ganzory et al., 2014).

Due to underreporting of incidents of sexual harassment towards nursing students and nurses, it is difficult to grasp the scope of the issue, and therefore this contributes to the lack of knowledge and implementation of policies and procedures that target sexual harassment in the clinical environment (Birks et al., 2018). Two quotes from the literature three decades apart refer to sexual harassment as “part of the job”, and a “working standard” (Perry, 1990, p.1; Hallett et al., 2021) suggesting there remains an imperative to operationalise expectations of a safe work environment for nurses. While Chapman (1993) reminds us that nurses are protected by law and formal processes, a recent UK study reported that 81% of participants had experienced nonphysical sexual violence on clinical placement in the past year (Hallett et al., 2021). Education about sexual harassment in preregistration nursing programmes would assist in recognition of the behaviour, and improve institutions' ability to respond, build resilience and improve coping mechanisms (Chang et al., 2021; El‐Ganzory et al., 2014; Kim et al., 2018). An additional need for training of people in positions of leadership, including facilitators and academics was also noted (Budden et al., 2017; Cogin & Fish, 2009; Finnis & Robbins, 1994), with strong, supportive leadership decreasing the prevalence of sexual harassment (Cogin & Fish, 2009), with likely flow‐on benefits to student nurses.

## 5 DISCUSSION

This literature review examines what is known about nursing students' experiences of sexual harassment on clinical placements. The prevalence of sexual harassment among student nurses occurs at a significant rate that should be cause for concern. Major contextual influences include nursing stereotypes, power imbalances present in the clinical setting, nursing work and a lack of coordinated responses to sexual harassment. While the higher rates of female students experiencing sexual harassment are likely reflective of the gender ratio among participants, and within nursing, this can both reinforce and mask the pervasiveness of gendered violence in female‐dominated professions which is influenced by the types of jobs (and the level of power) held by men and women (Durana et al., 2018).

Conceptually, the inequitable relations of power and their effects on sexual harassment can be seen as an overarching theme within which subthemes can be identified: gender influences; under‐reporting; nursing stereotypes; and the consequences of sexual harassment. The “power‐threat model” is a gender‐based hypothesis explored by McLaughin et al. (2012) who pose that women who threaten men's dominance are more likely to be victims of sexual harassment. This threat is particularly relevant for nurses and nursing students as they take on the role of the carer, breaking social and cultural boundaries regarding the body (Lawler, 1991). Lawler's (1991) influential work explores the experiences that nursing students have when first breaking social norms surrounding body care, and how students new to such care often feel awkward and embarrassed. The students' awkwardness within these moments draws closer attention to their inexperience and junior position during clinical placement, and thus sets conditions for exploitation and may thereby increase the risk of sexual harassment (Ferns & Meerabeau, 2008; Lawler, 1991).

From Halloween costumes to popular television shows, the sexualisation of nurses remains strong within contemporary discourse. Galdi et al. (2014) explore how the general sexualisation of women in the media alters individual consumers' perceptions of women and influences the likelihood of sexual harassment. The sexualisation of women (and nurses) in popular culture promotes the constitution of women as sexual objects, increasing the prevalence of sexual harassment in society and the workplace (Galdi et al., 2014), and in a focussed way for nursing students.

The COVID‐19 pandemic has internationally intensified social constructions of nurses as “angels” and “heroines”, perpetuating the gendered nature of the nursing role and the desirability of nurses, further increasing the risk of sexual harassment of nursing students (Lee et al., 2011; Stokes‐Parish et al., 2020). The trope of nurses as angels entrench the ideas of the profession and work as feminine (Stokes‐Parish et al., 2020). While the “heroine” figure does not follow the dominant discourse of femininity, heroism perpetuates self‐sacrificing ideas, where nurses are socialised to take risks, invoking conceptions of courage (Einboden, 2020). The nature of these tropes undermines nursing students' power and positions them at a higher risk of workplace violence, increasing their risk of sexual harassment (Einboden, 2020; Lee et al., 2011; Stokes‐Parish et al., 2020). Fedele (2019) states that although the common altruistic and caring image of the nurse positively contributes to the community's trust in the profession, this also creates misinformation about the nurses' role. Fedele (2019) supports the findings of this review that contemporary workplace hierarchies continue to position nurses as subservient to doctors. By undermining nurses' autonomy, the belief that nurses are subservient to doctors is accentuated, thus cementing nursing students' powerlessness in the clinical placement setting (Fedele, 2019).

The topic of sexual harassment for nurses and nursing students is extremely relevant in today's sociopolitical climate. The American Nurses Association recently addressed the issues of abuse towards nurses, including sexual harassment through a social initiative “#EndNurseAbuse” (American Nurses Association, 2018). In 2020, the Australian Health Practitioner Regulation Agency, NSW Nurses and Midwives Association, and Australian College of Nursing (ACN) all released position statements urging healthcare staff to report incidents of workplace sexual harassment, and through doing so highlighted the need for change. The ACN notes the need for culture change surrounding the way sexual harassment in the workplace is viewed, reported and understood (ACN, 2020). The call for improved workforce training in this statement and throughout this literature review prompts questions about how effective educational programmes are in combating sexual harassment. Confirming the findings of this literature review, a recent survey of surgical trainees did not report sexual harassment due to the assumptions that reporting would be a waste of time that perpetrators had no malicious intent, and due to fear of repercussions (Freedman‐Weiss et al., 2020).

Freyd and Smidt (2019) distinguish the need for education rather than training, on the basis that the implementation of educational programmes that involve critical thinking and the building of a complex understanding are more effective than training with simple online modules. Roehling and Huang (2018) concur, finding that education alone is not enough for the prevention and reduction of workplace sexual harassment; but that skill development and analysis of workplace behaviours are also required. Other effective implementations include bystander‐intervention training and manager training (Dobbin & Kalev, 2020).

Internationally, specific student services have been developed to support students in managing sexual violence they encounter on campus or within the context of their university lives. For example, in 2019, the University of Sydney implemented a specific service: “Safer Communities on Campus” (University of Sydney, n.d.) with a mandate to support the management of sexual violence and to mitigate harm for the many students who are affected. In north America, similar services are offered, including the following: “Sexual Harassment and Assault Response & Education” (SHARE) (Yale University, n.d.), and the “Sexual Violence Prevention & Support Centre” (University of Toronto, n.d.). Both provide crisis support, professional counselling and referrals for students who have experienced sexual violence.

While nursing students are more likely to be affected by sexual harassment than many other healthcare workers, a better understanding of nursing students' experiences could support the development of meaningful responses. Of the 26 papers included in this review, only 10 were published within the last five years (see Tables 2 and 3). The findings of this review support the notion that there is limited specific knowledge regarding nursing students' experiences of sexual harassment, including how they choose to report incidents or the responses they receive. Further research is needed to establish nursing students' experiences of sexual harassment on clinical placement in the current, contemporary social climate. Although emerging professional/national bodies are attempting to address the problem, understanding to what extent nursing students are “slipping through the cracks” is urgent, due to their short appearances and lack of confidence in the clinical setting.

### 5.1 Limitations

The practice of prospectively registering reviews is increasingly encouraged. This allows scrutiny of the consistency between the planned search strategy and the reported search strategy to ensure that the methods hold integrity, and have not been changed to suit the agenda of the researcher. The methods protocol for this review was not prospectively registered, and therefore such scrutiny is not possible.

Given the nursing focus of this review, only three databases were accessed, including CINAHL Complete, a nursing‐focussed database. While the PRISMA 2020 item checklist (Page et al., 2021) does not specify a minimum number of sources, increasing the number of databases searched would strengthen confidence in the study's comprehensiveness. As grey literature often is not contained in major databases, applying systematic methods of searching such papers is difficult, and therefore leaves room for missed literature, as does the inclusion of papers only in the English language. Initial screening was conducted by a single researcher, and this increases the possibility of missed article inclusion or inappropriate exclusion.

## 6 CONCLUSION

Internationally, a culture change is required that reflects broader social understandings of sexual violence for sexual harassment to be meaningfully addressed in the clinical setting. Nursing students are in a particularly vulnerable position due to gender inequity, their temporary position in a clinical area and limited expertise. They are also hindered by a sense of perceived vulnerability to patient complaints and the impact it may have on their success in the degree programme. Although there is increasing attention from professional nursing organisations, through position statements and procedures to protect nurses in the clinical setting, whether these measures offer effective protection for today's nursing students is yet to be determined.

## 7 RELEVANCE TO CLINICAL PRACTICE

The findings of this literature review are relevant not only to nursing students, but nurses, other health professionals, and students at all levels of education. Recommendations for clinical practice are transferable across care settings. The current evidence demonstrates that education is a strong protective factor for individuals experiencing sexual harassment in the workplace. Change to formal curriculum, the introduction of hospital‐based sexual harassment training as well as online modules related to workplace sexual harassment could have a positive impact on student experience and safety. Addressing barriers to reporting incidents of sexual harassment also promises to support a realistic understanding of the scope of the problem. Furthermore, the development of safe contexts to engage in dialogue and open conversations regarding experiences of sexual harassment in the workplace could support collective action to develop appropriate responses.

## FUNDING INFORMATION

This research is not funded.

## CONFLICT OF INTEREST

There is no perceived conflict of interest from any authors.

## Data Availability

Data sharing not applicable to this article as no datasets were generated or analysed during the current study.
